# 15-year remission in refractory FLT3-mutated AML attained by sorafenib

**DOI:** 10.1007/s00277-024-06012-3

**Published:** 2024-09-25

**Authors:** Christoph Rummelt, Dietmar Pfeifer, Ralph Wäsch, Robert Zeiser, Justus Duyster, Jürgen Finke, Michael Lübbert

**Affiliations:** Department of Hematology, Oncology and Stem Cell Transplantation, Faculty of Medicine, University Medical Center Freiburg, University of Freiburg, Freiburg, Germany

**Keywords:** Acute myeloid leukemia, FLT3-ITD, Sorafenib, Donor lymphocyte infusion, WT1

The outcome of most AML patients relapsing after allogeneic hematopoietic cell transplantation (HCT) remains dismal. The FLT3 internal tandem duplication (ITD) mutation provides a rational therapeutic target for treatment with tyrosine kinase inhibitors (TKIs) in the post-HCT setting. Sorafenib is widely used as maintenance therapy and induces durable remissions in patients relapsing after HCT in combination with donor-lymphocyte infusions (DLIs) via sorafenib-induced upregulation of IL-15 in FLT3-ITD-mutated blasts [1, 2]. We report a remarkable course of a patient who is in continued molecular remission following 7.5 years of sorafenib treatment (combined with a total of 13 DLIs) and 7.5 years without any specific anti-leukemic therapy.

In December 2008 the 38 year-old patient presented with *de novo* AML, normal karyotype, with FLT3-ITD (high), NPM1D, a DNMT3A and a WT1 R462W exon 9 hotspot mutation (Fig. 1, suppl. material). She was treated within the RATIFY trial [3], receiving two inductions and one consolidation plus study drug, but was chemotherapy-refractory (15% bone marrow blasts). HCT was performed after BU/CY conditioning, but already 5 weeks later, she presented with full-blown progression (WBC 76 000/µl). Molecular genetics revealed the mutations from initial diagnosis with similar variant allele frequencies (VAFs) except for the WT1 mutation (VAF almost doubled, from 48 to 88%), in line with a newly acquired loss of the WT1 wildtype (WT) allele (on chr. 11p13) by a small interstitial deletion on chromosome 11, bands p11p15. Cyclosporine was stopped and sorafenib (2 × 400 mg/day) resulted in immediate, massive cytoreduction. Treatment was continued for 10 weeks but measureable residual disease (MRD) persisted, which was eliminated after three DLIs. MRD negativity was maintained with continued sorafenib and 11 further DLIs over a total of 7.5 years. Subsequently, treatment was stopped and the unmaintained MRD negative CR persists until today (Fig. 1). We hypothesize that this ongoing long-term remission results from an interaction between the driver mutations with sorafenib and DLIs. Sole expression of the mutated WT1 allele might have rendered the blasts more susceptible to the sorafenib-enhanced GvL effect, especially when boosted with DLIs. The mechanistic role and the role as a predictor of response of mutated WT1 in this context is unclear. Analysis of the WT1 mutational status in larger cohorts [4, 5] treated with allogeneic HCT and might reveal a correlation to better response.

**Fig. 1:**
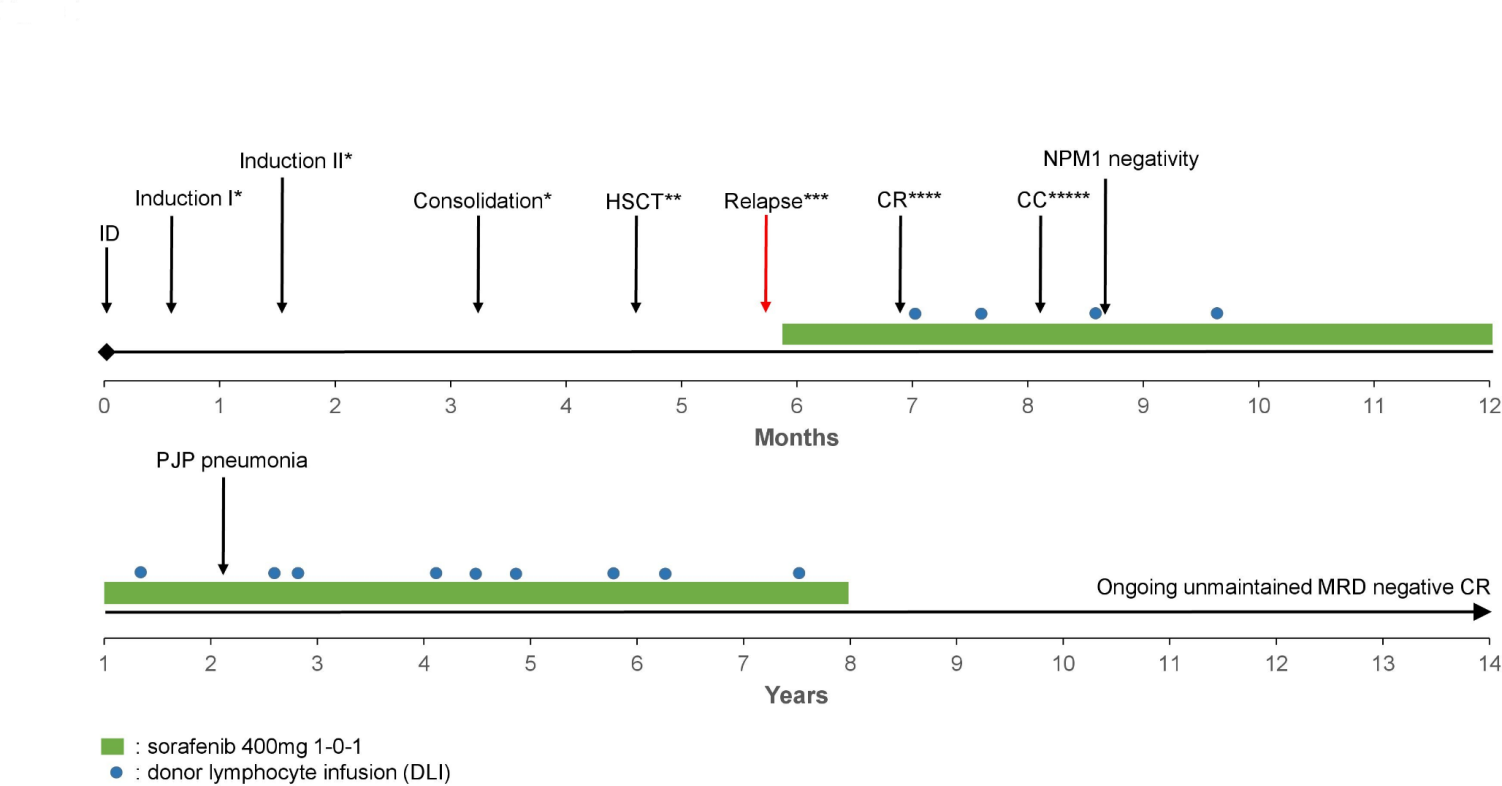
Clinical course of a primary refractory FLT3-ITD-positive AML patient achieving a durable remission with a combination of sorafenib and donor lymphocyte infusions. The patient (38 year-old female) was diagnosed with AML with the following mutations: FLT3-ITD (high), NPM1D, DNMT3A and WT1 R462W. Therapy was initiated within the RATIFY trial with two 7 + 3 (+ placebo) inductions and one high-dose cytarabine consolidation, but was refractory with 15% BM blasts hereafter (*). HSCT from an HLA matched unrelated male donor was conducted after BuCy conditioning, and GvHD prophylaxis was performed with 10 mg campath (2x), cyclosporine and MM (**). She experienced full-blown hematologic progression only 5 weeks after HCT, so cyclosporine and MMF were stopped. At that time FLT3-ITD and NPM1 were positive and a small interstitial deletion on chromosome 11, bands p11p15 was acquired (***). Sorafenib 400 mg 1-0-1 was initiated which led to CR with mixed chimerism and NPM1 persistence (****). After two DLIs, she achieved a complete chimerism and a NPM1 3 log decrease (*****). After one more DLI, MRD was negative with no more detectable NPM1. Therapy was stopped after 7.5 years on continued sorafenib therapy and 10 additional DLIs. Since then, she remains in ongoing unmaintained MRD negative CR (7.5 years until today)

Sorafenib and DLIs can induce long-lasting molecular remissions in patients with FLT3-ITD mutations relapsing or progressing after HCT. We speculate that selection for mutated WT1 may have contributed to the exceptional response to this combined treatment in our special case (see also suppl. material for further discussion). We want to motivate others to embrace the concept of continued FLT3 inhibition enhanced with donor lymphocyte infusions in case of active FLT3-ITD positive AML after HCT.

## Electronic supplementary material

Below is the link to the electronic supplementary material.

Supplementary Material 1

## Data Availability

No datasets were generated or analysed during the current study.
