# Supplementary material for: 15-year remission in refractory FLT3-mutated AML attained by sorafenib
Source: Ann Hematol. 2024 Sep 25;103(11):4801–3. doi: 10.1007/s00277-024-06012-3 (PMC11534974; doi:10.1007/s00277-024-06012-3)
Supplement: Supplementary file 1 — Supplementary Material 1 [file 277_2024_6012_MOESM1_ESM.docx]

**Supplementary Material**

**Methods:**

Myeloid Panel (MP): The Illumina TruSight Myeloid sequencing panel was used for targeted resequencing and processed as described by the manufacturer (Illumina Inc., San Diego, CA). FASTQ-files were further analyzed with the SeqNext software (JSI Medical Systems, Ettenheim, Germany). We used a significance threshold of 2% for the detection of missense mutations, with a minimum coverage of 100 reads and 50 reads per variant. Sequencing was done on an Illumina MiSeq. Karyotyping was performed at the MLL laboratories (Munich, Germany).

**Mutations at diagnosis:**

FLT3-ITD: 66 base-pair insertion at exon 14 (VAF 19%)

NPM1: NPM1D (VAF 40%)

DNMT3A: Deletion at D391Mfs*16 (COSM7349596) of yet unknown clinical significance (VAF 43%)

WT1: R462W (COSM21417, exon 9 hot-spot mutation, VAF 48%)

**Full karyotype:**

46,XX,del(2)(q31q34),der(11),inv(11)(p15q14),del(11)(p11p15) [20]

**HCT data:**

HCT was performed with standard myeloablative Bu/Cy conditioning and peripheral blood stem cells from a matched unrelated donor. Graft versus host disease (GvHD) prophylaxis was performed with alemtuzumab 10mg for 2 days, mycophenolate mofetil 500mg BID and cyclosporine A with serum plasma level target of 150-200ng/ml. She never developed acute or chronic GvHD.

**Supplementary discussion:**

Treatment of FLT3-ITD mutated patients that relapsed after allogeneic HCT with the TKI sorafenib has proven to be a feasible concept by Metzelder et al.^1^ They postulated that the successful treatment with sorafenib requires the additional graft versus leukemia (GvL) effect of an allogeneic HCT since sorafenib does not induce durable remissions in de novo AML.^2^ In their retrospective analysis, molecular complete remission (MCR) was the only predictor for long-term response of these patients. Our patient was part of this analysis (#2) and is an instructive example for such a treatment success. However, our patient was the only one to receive DLIs after the initiation of sorafenib treatment in this cohort and achieved MCR only hereafter (Fig. 1). Data from our institution by Mathew et al. support this observation, since sorafenib induced IL-15 production in FLT3-ITD mutated AML blasts CD8 positive T-cell response from allogeneic T-cells.^3^ Unfortunately, data on IL-15 induction in blasts of our patient is not available. Another effort by Sharma et al. did not result in long-term remissions in these patients^4^ but none of them was treated with additional DLIs. Hence, we postulate that a successful salvage of these patients with sorafenib requires the addition of DLIs. Of note, CR1 before allogeneic HCT was not achieved in our patient and she experienced a very early and pronounced hematologic progression, demonstrating the strong effect that the combination of sorafenib and DLIs in the context of an allogeneic HCT can have in an otherwise dismal situation.

Our patient was treated within the RATIFY trial^5^ and, as we now know after unblinding, did not receive the study drug midostaurin during induction therapy. Standard of care today however is the treatment with midostaurin during induction and before HCT. It is therefore debatable if our patient would have progressed with a FLT3-ITD positive clone and if so, if a salvage therapy with sorafenib and DLIs would have achieved such a treatment success.

Apart from achieving MCR, other predictors of treatment success with this regimen need to be distinguished. In our patient, the loss of the WT1 wild type allele at relapse after allogeneic HCT suggests that the WT1 R462W mutation was selected during clonal evolution. We speculate that sole expression of the mutated WT1 allele rendered the blasts more susceptible to the sorafenib-enhanced graft versus leukemia (GvL) effect, especially when boosted with DLIs. The mechanistic role and the role as a predictor of response of mutated WT1 in this context remains to be elucidated. Revision of the WT1 mutational status in larger cohorts treated with allogeneic HCT and sorafenib (e.g. the SORMAIN population^6^) might reveal a correlation to better response.

The treatment duration of sorafenib and DLIs that is needed to accomplish durable, unmaintained remission is unknown. Achieving MCR is mandatory and maintaining it for several years is arguably necessary. Discontinuation is feasible, as Metzelder et al. also demonstrated.^1^ Continued immunosuppression and toxicities are also strong arguments to wage discontinuation as our patient experienced PJP pneumonia and prolonged bleeding after conization, which eventually led to discontinuation of the treatment. We therefore also argue to continue anti-infective prophylaxis during sorafenib treatment.

With regards to the recent report by Levis et al. on gilteritinib as maintenance therapy after HCT,^7^ we were also able to demonstrate the ability of gilteritinib to upregulate IL-15 in FLT3-ITD-mutated blasts,^3^ indicating that it could fulfill the same role as sorafenib with suspected superior toxicity profile. If the greater target-specificity renders gilteritinib superior to sorafenib remains to be elucidated. Also, the treatment duration with gilteritinib and addition of DLIs to the regimen remain questions to be answered.

Taken together, sorafenib and DLIs can induce long-lasting molecular remissions in patients with FLT3-ITD mutations relapsing after allogeneic HCT. We speculate that mutated WT1 may have contributed to the exceptional response to this treatment combination.

**Literature of supplementary discussion:**

1. Metzelder, S. K. *et al.* Long-term survival of sorafenib-treated FLT3-ITD-positive acute myeloid leukaemia patients relapsing after allogeneic stem cell transplantation. *Eur. J. Cancer Oxf. Engl. 1990* **86**, 233–239 (2017).

2. Borthakur, G. *et al.* Phase I study of sorafenib in patients with refractory or relapsed acute leukemias. *Haematologica* **96**, 62–68 (2011).

3. Mathew, N. R. *et al.* Sorafenib promotes graft-versus-leukemia activity in mice and humans through IL-15 production in FLT3-ITD-mutant leukemia cells. *Nat. Med.* **24**, 282–291 (2018).

4. Sharma, M. *et al.* Treatment of FLT3-ITD-positive acute myeloid leukemia relapsing after allogeneic stem cell transplantation with sorafenib. *Biol. Blood Marrow Transplant. J. Am. Soc. Blood Marrow Transplant.* **17**, 1874–1877 (2011).

5. Stone, R. M. *et al.* Midostaurin plus Chemotherapy for Acute Myeloid Leukemia with a FLT3 Mutation. *N. Engl. J. Med.* **377**, 454–464 (2017).

6. Burchert, A. *et al.* Sorafenib Maintenance After Allogeneic Hematopoietic Stem Cell Transplantation for Acute Myeloid Leukemia With FLT3-Internal Tandem Duplication Mutation (SORMAIN). *J. Clin. Oncol. Off. J. Am. Soc. Clin. Oncol.* **38**, 2993–3002 (2020).

7. Levis, M. J. *et al.* Gilteritinib as Post-Transplant Maintenance for AML With Internal Tandem Duplication Mutation of FLT3. *J. Clin. Oncol. Off. J. Am. Soc. Clin. Oncol.* **42**, 1766–1775 (2024).
